# Citizen-Led Intervention to Enhance Social Engagement of Older Adults in Depopulated Rural Areas in Korea

**DOI:** 10.1093/geroni/igaf122.3427

**Published:** 2025-12-31

**Authors:** Suhyeon Choi, Susan Park, Sunyoung Park, Soong-nang Jang

**Affiliations:** Chung-Ang University, Seoul, Republic of Korea; Inha University Medical School, Incheon, Republic of Korea; Chung-Ang University, Seoul, Republic of Korea; Chung-Ang University, Seoul, Republic of Korea

## Abstract

Background This study evaluated the effectiveness of a citizen-led intervention designed to enhance social engagement and improve health among older adults in the depopulated areas of Jeongeup City, Korea. These areas face population aging, economic stagnation, and limited infrastructure, increasing healthcare and social service needs. Method The citizen-led intervention included 1) assessing the care needs of older adults, 2) coordinating necessary healthcare and social welfare services accordingly, and 3) implementing a 12-week group frailty management program at community centers. Baseline and follow-up measures were conducted in November 2023 and August 2024, respectively, among older adults aged 65 years and older. Social engagement, subjective health status (SHS), and care fulfillment were measured. The treatment and control groups were conveniently assigned 157 and 2,474 participants, respectively. To ensure group comparability, propensity score matching (1:3) was performed based on demographic and health factors (age, sex, disability, economic status, the number of chronic diseases, etc.), resulting in 267 matched control participants. Results Social engagement significantly increased in the treatment group (β = 0.24, p = 0.016). Care fulfillment was significantly higher in the treatment group (M = 8.34, SD = 1.61) than in the control group (M = 6.11, SD = 1.81, p < 0.01). SHS declined over time (OR = 0.54, p < 0.01), with no significant difference between groups. Conclusion Citizen-led interventions improved social engagement and care fulfillment but had limited effects on health outcomes. Trained community members may play a role in addressing care needs by organizing local support networks.

